# Neutrophil to lymphocyte ratio in odontogenic infection: a systematic review

**DOI:** 10.1186/s13005-024-00421-5

**Published:** 2024-03-28

**Authors:** Saeideh Ghasemi, Bardia Mortezagholi, Emad Movahed, Sahar Sanjarian, Arshin Ghaedi, Amirhossein Mallahi, Aida Bazrgar, Monireh Khanzadeh, Brandon Lucke-Wold, Shokoufeh Khanzadeh

**Affiliations:** 1 Dental school, Tabriz University of Medical Sciences, Tabriz, Iran; 2 Dental Research Center, Faculty of Dentistry, Islamic Azad University of Medical Sciences, Tehran, Iran; 3 Islamic Azad University of Medical Sciences, Tehran, Iran; 4 Student Research Committee, School of Medicine, Shiraz University of Medical Sciences, Shiraz, Iran; 5 Trauma Research Center, Shahid Rajaee (Emtiaz) Trauma Hospital, Shiraz University of Medical Sciences, Shiraz, Iran; 6 Geriatric & Gerontology Department, Medical School, Tehran University of medical and health sciences, Tehran, Iran; 7 Department of Neurosurgery, University of Florida, Gainesville, USA; 8 Tabriz University of Medical Sciences, Tabriz, Iran

**Keywords:** Neutrophil to Lymphocyte Ratio, Inflammation, Odontogenic infection, Systematic review

## Abstract

**Background:**

We conducted this systematic review to compile the evidence for the role of neutrophil to lymphocyte ratio (NLR) in odontogenic infection (OI) and to determine whether NLR is elevated in patients with OI. This was done to aid physicians in better understanding this condition for clinical management.

**Methods:**

The search was conducted on PubMed, Scopus, and Web of Science libraries on March 30, 2023. Two reviewers independently screened the studies using Endnote software. The Newcastle-Ottawa Scale (NOS) was used to evaluate the quality of the studies.

**Results:**

A total of nine studies were included in the review. Among patients with OI, positive and statistically significant correlations of NLR were seen with more severe disease, a prolonged hospital stay, postoperative requirement of antibiotics, and total antibiotic dose needed. In the receiver operating characteristics (ROC) analysis, the optimum cut–off level of NLR was 5.19 (specificity: 81, sensitivity: 51). In addition, NLR was correlated with preoperative fever (*p* = 0.001). Among patients with Ludwig’s Angina, NLR could predict disease severity and length of stay in the hospital (*p* = 0.032 and *p* = 0.033, respectively). In addition, the relationship between the NLR and mortality was statistically significant (*p* = 0.026, specificity of 55.5%, and sensitivity of 70.8%). Among patients with severe oral and maxillofacial space infection, a positive correlation was found between IL-6 and CRP with NLR (rs = 0.773, *P* = 0.005 and rs = 0.556, *P* = 0.020, respectively). Also, a higher NLR was considered an essential predictor of organ involvement (*P* = 0.027) and the number of complications (*P* = 0.001). However, among diabetes mellitus (DM) patients afflicted with submandibular abscesses, NLR had no association with therapeutic response.

**Conclusions:**

Many people around the world suffer from OI, and a cheap and fast biomarker is needed for it. Interestingly, inflammation plays a role in this infection, and elevated NLR levels can be a good biomarker of inflammation and, as a result, for OI progression.

## Background

Generally, it is thought that the incidence of severe odontogenic infections (OI) is decreasing due to several factors. These include advancements in healthcare delivery, availability of antimicrobials, and general improvements in oral hygiene [1, 2]. Dental infections are rather common, with some studies stating that they account for many antibiotic prescriptions. However, if left untreated, they may spread to the maxillofacial and cervical areas, offering worsened possible complications [3, 4].

Identifying individuals with OI who are more likely to have severe outcomes is critical when treating them. These findings may impact dose and treatment efficacy decisions in certain difficult instances. One key prognostic factor is the intensity of the immunoinflammatory response [5]. Numerous ratings that indicate the severity and duration of infections based on parameters collected from basic blood tests have been created [6]. Those characteristics would be particularly useful owing to their ease of availability and low cost. Neutrophil to Lymphocyte Ratio (NLR), C-reactive protein (CRP), and white blood cell (WBC) counts are a few examples of objective evaluation parameters that have been studied; however, the results have been inconsistent [7–9].

NLR is widely available, easily calculated, simple, and sensitive but not highly specific biomarker of inflammation and stress [10]. NLR has been used to predict illness outcomes in cardiovascular disease, cancer, inflammatory bowel disease, and renal disease [11–13]. Although being regularly used in almost all medical specialties today, including surgical fields, emergency care, and infections of the craniofacial region, surprisingly, few studies have been done on this ratio for OI [14]. In individuals who have aberrant inflammatory reactions, NLR is frequently increased. Moreover, in response to systemic inflammation, lymphocytes are redistributed into the lymphatic system, resulting in lymphopenia [15]. This biomarker, which can be determined from a complete blood count, is a sign of active infection [16]. Since neutrophils are regarded as the first line of defense in the innate immune response, resulting in neutrophilia, an increased NLR in a patient with a deep neck space infection is probable.

Since OI is related to inflammation, investigating the NLR in this infection is warranted. If the link between elevated NLR and OI is established, costly tools and resources for diagnosing and treating this condition can be mitigated. We conducted a systematic review to determine the diagnostic and prognostic role of NLR in different aspects of OI, including the severity of the disease, signs, symptoms, length of stay in the hospital, postoperative doses of antibiotics, total antibiotic doses, therapeutic response, mortality, and organ involvement.

## Materials and methods

This systematic review was performed based on the Preferred Reporting Items for Systematic Reviews and Meta-Analyses (PRISMA) guidelines. We have registered this study in PROSPERO (CRD42024500767). We searched PubMed, Web of Science, and Scopus libraries on March 30, 2023, using keywords (“Neutrophil to lymphocyte ratio“[All Fields] OR “NLR“[All Fields]) AND (“dental“[All Fields] OR “Odontogenic“[All Fields]) AND (“Infection“[All Fields] OR “infections“[MeSH Terms]) with no date or language limitations. Studies attempting to determine the diagnostic role of NLR were checked. We found pertinent studies by manually searching references from all eligible studies, the Science Citation Index Expanded on Web of Science, review articles, and the top 50 citations for each paper using PubMed’s related articles’ feature.

Randomized controlled trials, retrospective or prospective cohort studies, and case-control studies assessing the use of NLR for the risk stratification or identifying OI. Studies including at least 20 participants were eligible. The outcome was NLR’s diagnostic and prognostic role in different aspects of OI, including the severity of disease, signs and symptoms, length of stay in the hospital, postoperative doses of antibiotics, total antibiotic doses, therapeutic response, mortality, and organ involvement. Exclusion criteria were: (1) experimental investigations; (2) articles containing overlapped data; (3) articles without complete data; (4) editorials, case series or case reports, abstracts, and review articles.

Two reviewers independently searched articles to evaluate their relevancy and methodological quality, and they chose all studies that were appropriate for review. Any disagreements about the inclusion or exclusion of articles were cleared up by discussion. The Newcastle-Ottawa Scale (NOS) was used to evaluate the risk of bias. If an article had a NOS score > 5, we defined it as a high-quality study. By using prepared data extraction forms, the following information was collected: [1] the first author’s name; [2] the country of origin; [3] the study design; [4] the year of publication; [5] the NLR level of participants; [6] the number of participants; [7] age; [8] gender; [9] comorbidities; and [10] statistical items including like P-value, odds ratio, best cut off value, and its sensitivity and specificity.

## Results

**Fig. 1:**
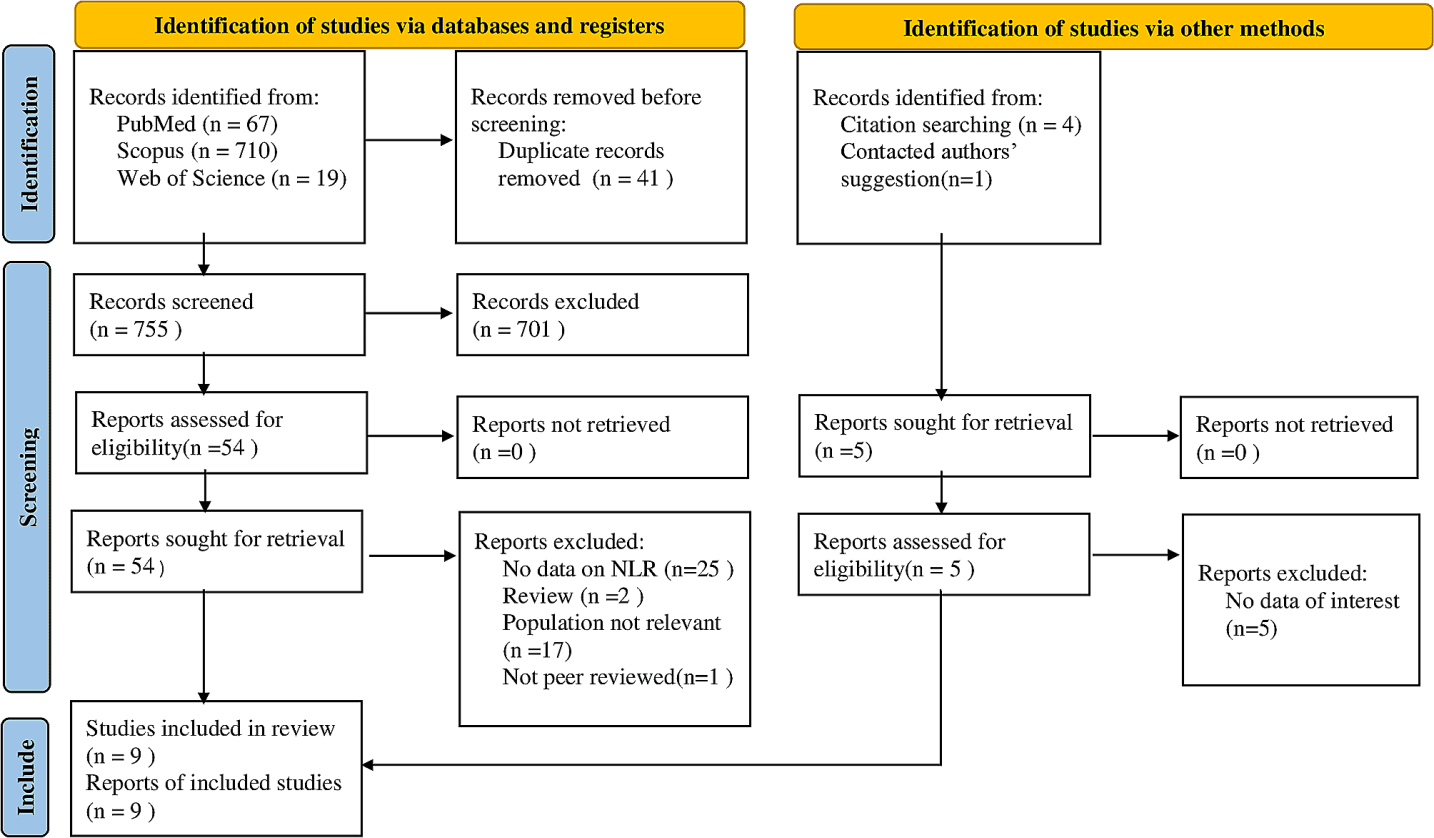
Flow diagram of includes searches

A total of 801 articles were identified through databases and manual searches. Finally, nine articles were included for this review. The article-chosen process is illustrated in Fig. 1. The selected articles comprised two prospective, six retrospective, and one cross-sectional study. The characteristics of the included studies and their risk of bias assessment are summarized in Table 1. One study had a NOS score of six, five had a NOS score of seven, and two had a NOS score of eight. All studies therefore met criteria for high quality.

**Table 1:** General characteristic of included studies

First author	Year	Region	Design	Sample size	Mean age	Gender (percentage of males)	Main result	NOS Score
Kusumoto et al. [19]	2022	Japan	Retrospective	Total: 271 Group I (cellulitis): 123 Group II (cellulitis with superficial abscess): 61 Group III (profound abscess): 74 Group IV (NSTI): 13	Total: 61 (41, 74.5) Group I: 56 (37, 71.5) Group II: 59 (40, 77) Group III: 65 (47.3, 73.8) Group IV: 73 (41, 75)	Total: 138 (50.9%) Group I: 60 (48.8%) Group II: 35 (57.4%) Group III: 38 (51.4%) Group IV: 5 (38.5%)	CRP + NLR was an effective tool for diagnosing NSTI and decision-making for CT scan performance.	7
Pavan et al. [18]	2020	Brazil	Prospective	Total: 50 Group1(LOS ≤ 3 days): 17 Group2(LOS > 3 days): 33	Group1: 30.1 ± 15.7 Group2: 32.0 ± 15.6	Group1: 11 (64.7) Group2: 22 (66.7)	NLR showed a statistically significant and positive correlation concerning LOS.	6
Silva et al. [24]	2022	Brazil	Prospective	Total: 66	Total: 32.7	a ratio of 1.4:1, with a predominance of male sex	Higher NLR values were significantly associated with greater involvement of fascial regions.	7
Chamora et al. [25]	2021	Indonesia	Retrospective	Total: 15 Group1(with DM): 6 Group2(without DM): 9	Group1: 50.33 ± 9.54 Group2: 33.56 ± 17.78	Group1: 3 (50) Group2: 6 (66.7)	The mean NLR of patients with DM was lower than patients without DM, and there was no significant association between NLR and therapeutic responses.	7
Dogruel et al. [17]	2017	Turkey	Retrospective	Total: 93 Group1(LOS ≤ 1 days): 44 Group2(LOS > 1 days): 49	Total: 14.50 (7.50–32.00) Group1: 13.00 (8.00–32.00) Group2: 14.00 (7.00–30.00)	Total: 49 (52.1) Group1: 24 (53.3) Group2: 25 (51.0)	NLR was positively and statistically correlated with prolonged LOS, postoperative doses of antibiotics, and also total antibiotic doses.	8
Sakarozi et al. [20]	2022	Indonesia	Retrospective	Total: 96	NA	Total: 60 (62.5)	Significant relationships were observed between NLR and severity, LOS, and mortality in Ludwig’s angina patients.	7
Gallagher et al. [23]	2021	England	Retrospective	Total: 161 Group1(LOS ≤ 2 days): 91 Group2(LOS ≥ 3 days): 70	Total: 38.4 ± 16.8 (5–86) Group1: 36.1 (5–78) Group2: 41.4 (8–86)	Total: 89 (52.7) Group1: 41 (45.1) Group2: 48 (68.6)	NLR can be utilized as a prognostic modality in patients with OI. Patients with NLR ≥ 4.65 are more likely to be hospitalized for two days or more.	7
Rosca et al. [22]	2022	Romania	Retrospective	Total: 108 Group1(low-severity infection): 54 Group2(high-severity infection): 54	Group1: 46.7 ± 17.9 (18–81) Group2: 51.7 ± 18.1 (20–85)	Group1: 30 (55.6) Group2: 36 (66.7)	NLR values observed no statistical significance between groups. However, in logistic regression analysis adjusted for age, gender, and comorbidities, NLR reached statistical significance.	8
Xiaojie et al. [21]	2021	China	Retrospective	Total: 18	Total: 54 (16–75)	Total: 12 (66.6)	NLR was positively correlated with the number of organ involvement.	7

NSTI: necrotizing soft tissue infection, CRP: C-reactive protein, NLR: neutrophil to lymphocyte ratio, LOS: length of stay, DM: Diabetes mellitus, NA: not available, OMSI: oral and maxillofacial space infection

In a research published by Dogruel et al. [17], 100 patients with severe OI were retrospectively reviewed to assess the prognostic value of NLR and mean platelet volume (MPV). Based on the length of hospital stay, they divided patients into two groups: Group 1 with a hospital stay of one day or less and Group 2 with more than one day. No statistically significant difference was detected in age and gender (*p* = 0.925 for age and *p* = 0.823 for gender). Spearman’s correlation results indicated positive and statistically significant correlations of NLR with a prolonged hospital stay, postoperative antibiotics, and total antibiotic doses. In receiver operating characteristics (ROC) analysis, NLR’s optimum cut–off level was 5.19 (specificity: 81, sensitivity: 51). NLR of group 2 was detected to be 5.19 or higher. Patients were also divided according to the presence of fever, defined as a temperature of 38 °C or more pre- and post-operatively. As a result, NLR was correlated with preoperative fever (*p* = 0.001).

Pavan et al. [18] implemented a prospective research of 50 cases diagnosed with OI. They aimed to detect the value of vital sign changes and laboratory tests in determining the severity and length of stay (LOS). The authors categorized patients into group 1 and group 2, representing the hospital stays that lasted up to three days and more than three days, respectively. The distribution of cases regarding mean age and sex showed no statistical significance. The average LOS was 2.8 and 6.9 days for groups 1 and 2, respectively. Obtained mean values of all laboratory tests showed statistical significance between groups, apart from ESR (*p*-value of NLR = 0.044). Spearman’s correlation analysis revealed a positive correlation of LOS with NLR (r = + 0.291, *p* = 0.041), leukocytosis (r = + 0.284, *p* = 0.045), neutrophilia (r = + 0.302, *p* = 0.033), CRP levels (r = + 0.426, *p* = 0.003), and heart rate (r = + 0.311, *p* = 0.028). Moreover, the simple regression analysis of the numbers obtained in decreasing order demonstrated that 29.0% of the variation in hospital stay time was related to an increase in NLR.

Kusumoto et al. [19] retrospectively analyzed 271 patients with severe OI to evaluate the efficiency of routine blood tests as an early detective method. Patients were divided into four groups: cellulitis, cellulitis with superficial abscess formation, profound abscess formation, and necrotizing soft tissue infection (NSTI), representing groups I to IV, respectively. There was no significant difference concerning age and gender among these four groups (*P* = 0.087 for age and *P* = 0.561 for gender). There was an increasing pattern in all inflammatory and hematologic factors, including WBC, neutrophil, CRP, NLR, CRP + NLR, platelet-to-lymphocyte ratio (PLR), systemic immune-inflammation index (SII), and the Laboratory Risk Indicator for Necrotizing Fasciitis (LRINEC). This was consistent for group I to group IV (*P* < 0.001), independent from hemoglobin and lymphocyte fraction that tends to decrease. NLR was utilized to calculate SII (platelet count × neutrophil count/lymphocyte count). Moreover, the necessity for contrast-enhanced CT (CECT) was decided based on comparing these parameters between groups I + II and III + IV. They performed a decision tree analysis considering higher amounts of inflammatory and hematologic parameters in groups III + IV than in groups I + II. Decision-making for CECT and differentiation of group III + IV can be determined by SII of ≥ 282 or < 282 but with a CRP + NLR of ≥ 25 because of this analysis.

Sakarozi et al. [20] conducted a retrospective cohort study of 96 patients with Ludwig’s Angina, a submandibular cavity infection with cellulitis that can quickly progress and become life-threatening. They aimed to determine the association between pre-therapy NLR and various prognostic factors in these patients. Regarding gender distribution, males were more likely to develop the condition. ROC analysis illustrated the optimal cut-off value of 16.86 for NLR. Forty-nine samples were with low NLR (< 16.86), and 47 were with high NLR values (≥ 16.86). NLR reached a significant relationship in predicting disease severity and LOS (*p* = 0.032 and *p* = 0.033, respectively). This study evaluated mortality status during hospitalization. The relationship between NLR and mortality was statistically significant (*p* = 0.026, specificity of 55.5%, and sensitivity of 70.8%). Considering Kaplan Meier’s analysis, the low-NLR group’s survival rate was significantly higher than that of the high-NLR group (*p* = 0.009).

A retrospective study by Xiaojie et al. [21], including 18 patients with severe and extremely severe oral and maxillofacial space infection (OMSI), was carried out to determine the predictive value of NLR and IL-6 in OMSI intensity. Of these 18 patients, 12 had severe OMSI, and 6 had extremely severe OMSI. Correlation analyses demonstrated a positive correlation between IL-6 and CRP with NLR (rs = 0.773, *P* = 0.005 and rs = 0.556, *P* = 0.020, respectively). This study also revealed that the number of involved organs correlated positively with NLR (rs = 0.511, *P* = 0.030). Furthermore, the number of complications positively correlated with NLR values (*r* = 0.576, *P* = 0.012). Consequently, a higher NLR was considered an essential predictor of organ involvement (*P* = 0.027) as well as the number of complications (*P* = 0.001).

In another retrospective study, Rosca et al. [22] evaluated 108 hospitalized patients with OI to determine whether NLR and CRP were the accurate prognostic tools for OI severity. Considering the severity of the infection, cases were divided into two equal groups: Group A with mild to moderate infections; Group B with moderate to severe infections. Regarding age and sex, no statistically significant differences were found among groups A and B (*P* = 0.150 and *P* = 0.236, respectively). In contrast, coming from rural regions (*P* = 0.019), being afflicted with diabetes mellitus (*P* < 0.001), and being a smoker (*P* = 0.028) were more frequent in group B compared to group A. Regarding the infection characteristics among patients, abscesses comprise 70.4% of infections in group A, while associations of abscesses and cellulitis were responsible for 55.6% of infections in group B (*P* < 0.001). Moreover, group B had more patients developing sepsis than group A (*p*-value = 0.030). Severity evaluations were calculated according to SII and the Symptom Severity score (SS). SII and SS scores were significantly higher in group B patients (*P* < 0.001). Likewise, tested biomarkers, including the CRP-NLR association, showed higher scores in patients of group B (median score of 341.4 vs. 79.0 in group A, *P* < 0.001). No statistical significance prior to logistic regression was seen for NLR between groups A and B (*P* = 0.019). However, in logistic regression analysis adjusted for age, gender, and comorbidities, NLR reached statistical significance with an odds ratio of 4.46 (95% CI = (3.53–5.40), *P* < 0.001), and the CRP-NLR association accounted for a 7.28 (95% CI = (4.83–10.16), *P* < 0.001) higher risk for severe OI.

In parallel, Gallagher et al. [23] retrospectively enrolled 161 patients with deep neck space infections of odontogenic origin and divided them based on the LOS: Group 1 consisted of 91 patients admitted for zero to two days, Group 2 with 70 patients admitted for three or more days. The male gender was statistically significant (*p* = 0.03). In contrast, no statistical significance was found between groups regarding age evaluation (*p* = 0.47). The mean days of LOS were 2.9 ± 3.2 (range: 0.5–35), and the mean values of admission NLR were 7.5 ± 7.7. According to Spearman’s correlation test, admission NLR showed a positive correlation with LOS (*r* = 0.30, *p* ≤ 0.01). In the ROC analysis, the best cut-off value of NLR to predict a LOS ≥ two days was 4.65 (test specificity = 61.5%, test sensitivity = 61.4%). In addition, a cut-off of 11.75 for NLR predicted admission in ICU with 82.6% specificity and 66.7% sensitivity.

Silva et al. [24] prospectively collected 66 hospitalized patients with OI, aiming to detect the possible correlations between computed tomographic findings of involved fascial spaces and laboratory markers, length of hospital stays, and Intensive Care Unit (ICU) admission. The mean LOS was 4.3 days. The involvement of 240 fascial spaces was observed in this study, with a mean of 3.63 spaces per patient. Submandibular 65 (27.1%), buccal 50 (20.8%), and sublingual 44 (18.3%) were the first three involved spaces. Higher levels of neutrophils (*p* = 0.001), NLR (*p* < 0.001), and CRP (*p* < 0.001) were associated with more significant numbers of involved spaces. The mean LOS progressively increased regarding the number of involved fascial spaces. Also, the need for ICU admission and the number of fascial spaces revealed a significant difference (*p* < 0.001).

An analytic observational study by Chamora et al. [25] was performed to identify NLR correlation with LOS among two groups of patients with and without diabetes mellitus (DM) afflicted with submandibular abscesses. Of 15 patients, six were in the DM group, and nine were in the non-DM group. The mean age of DM patients was higher than non-DM participants (50.33 ± 9.54 years and 33.56 ± 17.78 years, respectively). In addition, the male-to-female ratio of DM patients was 1:1, compared to 2:1 for patients without DM. For DM participants, the mean length of hospital stay was 7.83 + 1.47, and for non-DM participants, it was 8.22 + 2.86. Considering the therapeutic response, assessments were based on LOS, defining a good response as LOS ≤ 7 days. The mean NLR of the non-DM group was higher than for patients with DM (16.53 ± 11 and 7.65 ± 4.92, respectively). However, this lower value in the DM group did not lead to a better therapeutic response. In this study, contrary to previous studies, the comparison of NLR between DM and non-DM patients for therapeutic responses did not reach statistical significance (*p* = 0.88 for the DM group and *p* = 0.5 for, respectively).

## Discussion

The main findings of our review are as follows: NLR had a significant and positive correlation with length of stay in hospital, disease severity, and the number of organ involvement.

As previously discussed, OI is one of the major dental and periodontal disorders worldwide, with significant financial impacts for patients [26]; as a result, an inexpensive and rapid biomarker is required to diagnose this disease efficiently and early. Notably, OI is influenced by inflammation, and high NLR values can be a helpful marker for inflammation. Therefore, it serves as a key contributor regarding active infection [19, 27].

Primarily, recognizing the relationship between NLR and this disease is related to a comprehensive understanding of the role of neutrophils and lymphocytes in OI. NLR is a primary ratio between regulatory immune cells, proinflammatory cells, neutrophils, and lymphocytes. Consequently, a higher NLR represents a higher level of inflammation, which can lead to infection development or severity. Neutrophilia and lymphocytopenia are indicators of the body’s response to bacterial infections. The NLR ratio, an effective biomarker for detecting bacterial infections, verified the link between inflammatory cells [28]. This ratio is less than or equal to 5 in a physiological setting, and when infection or severe inflammation is present, the value is greater than 6 [15].

Neutrophils are the most dominant leukocytes in natural immunity, and these types of cells increase under systemic inflammation. They are crucial components of the body’s response to bacterial infections and can phagocytize and kill microbes in the condition of infection. NLR values in infected patients predict bacteremia [20]. Baglam et al. revealed that patients with tonsillitis and deep neck space infection (DNSI) had higher values of NLR [29]. Therefore, NLR may be utilized as a simple, relatively cheap, and practical test to identify complications and complications in neck infections.

Previously, lymphocytopenia was accepted as an indicator of bacteremia but was not widely defined as an infection sign. Regarding the mechanism causing lymphocytopenia in sepsis and septic shock, the redistribution and margination of lymphocytes in the lymphatic system and apoptosis can be considered. Apoptosis is a condition that promotes sepsis. Persistent lymphocytopenia and neck infection risks result from lymphocyte apoptosis that develops rapidly in patients’ blood in septic shock [30]. Although multiple possible explanations exist, the underlying cause of the elevated NLR related to poor outcomes in patients with sepsis remains unknown. The physiological link between neutrophilia and lymphopenia during systemic stress and inflammation could be one of the plausible explanations. The growth in neutrophil production by the medulla and the reduction in the number of lymphocytes by apoptosis is due to insufficient eradication of the infectious nidus and persistent infection [31]. The severity of neutrophilia and lymphocytopenia in patients with systemic infection or inflammation closely correlates to the injury’s intensity, clinical status, and clinical outcome [15]. This admits that NLR can be applied as a diagnostic modality, assessing the infection intensity in patients with OI. Patients suffering from more intense infections and sepsis need prolonged hospitalizations. NLR values are expected to be used as diagnostic guidance in predicting patients afflicted with OI who may require a more extended stay in the hospital. NLR has been extensively studied as a test that can be a possible diagnostic tool for predicting mortality, host inflammatory responses, and chemotherapy responses [32–34]. One prospective cohort study identified high NLR as an independent prognostic modality of mortality in critically ill patients during the hospital stay and six months post-treatment [35]. Similarly, another study explained that the NLR value at presentation to the emergency room independently predicted 28-day mortality in cases with severe sepsis or septic shock [36]. Prior studies also demonstrated that first-admission NLR was significantly lower in patients who died before day 5 of a septic shock onset than those who survived [37, 38].

### Limitations

Our present study has several limitations that are worth mentioning. Primarily, the included studies in this review were limited by small sample sizes, leading to dissimilarities. Another relevant issue is that our findings may need more consistency to draw a concrete result. Studies with more participants will be essential to confirm the connection between NLR and OI. Another limitation was due to the retrospective design of most studies, as this type of study is dependent on medical records from the past, and the validity of these records is under debate.

### Conclusion

The results of our study support the link between NLR values and OI, and this ratio increases in affected patients. Our findings suggest that NLR could be a helpful modality that is easily obtained through a simple blood test. Early identification can aid in therapeutic interventions that tend to assist in preventing and treating OI and lowering long-term morbidity and mortality.

## Data Availability

All data generated or analysed during this study are included in this published article.

## Abbreviations

NLR: Neutrophil to lymphocyte ratio
OI: Odontogenic infection
CRP: C-reactive protein
WBC: White blood cell
NOS: Newcastle-Ottawa Scale
LOS: Length of stay
NSTI: Necrotizing soft tissue infection
LRINEC: Laboratory Risk Indicator for Necrotizing Fasciitis
CECT: Contrast-enhanced CT
OMSI: Oral and maxillofacial space infection
ROC: Receiver operating characteristics
ICU: Intensive Care Unit
DNSI: Deep neck space infection
DM: Diabetes mellitus

## References

[1] Alotaibi N, Cloutier L, Khaldoun E, Bois E, Chirat M, Salvan D (2015). Criteria for admission of odontogenic infections at high risk of deep neck space infection. Eur Annals Otorhinolaryngol head neck Dis.

[2] Bali RK, Sharma P, Gaba S, Kaur A, Ghanghas P (2015). A review of complications of odontogenic infections. Natl J Maxillofacial Surg.

[3] Sánchez R, Mirada E, Arias J, Paño Pardo JR. Burgueño García M. Severe odontogenic infections: epidemiological, microbiological and therapeutic factors. 2011. 10.4317/medoral.16995 20711116

[4] Blankson P-K, Parkins G, Boamah MO, Abdulai AE, Ahmed A-M, Bondorin S et al. Severe odontogenic infections: a 5-year review of a major referral hospital in Ghana. Pan Afr Med J. 2019;32(1). 10.11604/pamj.2019.32.71.17698 PMC6561007 31223362

[5] Stephens MB, Wiedemer JP, Kushner GM (2018). Dental problems in primary care. Am Family Phys.

[6] Ince N, Güçlü E, Sungur MA, Karabay O (2020). Evaluation of neutrophil to lymphocyte ratio, platelet to lymphocyte ratio, and lymphocyte to monocyte ratio in patients with cellulitis. Revista Da Associação Médica Brasileira.

[7] Shumilah AM, Othman AM, Al-Madhagi AK (2021). Accuracy of neutrophil to lymphocyte and monocyte to lymphocyte ratios as new inflammatory markers in acute coronary syndrome. BMC Cardiovasc Disord.

[8] Spoto S, Lupoi DM, Valeriani E, Fogolari M, Locorriere L, Beretta Anguissola G (2021). Diagnostic accuracy and prognostic value of neutrophil-to-lymphocyte and platelet-to-lymphocyte ratios in septic patients outside the intensive care unit. Medicina.

[9] Gurguş D, Grigoraş ML, Motoc AGM, Zamfir CL, Cornianu M, Faur CI (2019). Clinical relevance and accuracy of p63 and TTF-1 for better approach of small cell lung carcinoma versus poorly differentiated nonkeratinizing squamous cell carcinoma. Rom J Morphol Embryol.

[10] Miloro M, Ghali G, Larsen PE, Waite PD. Peterson’s principles of oral and maxillofacial surgery: Springer; 2004.

[11] Li M, Spakowicz D, Burkart J, Patel S, Husain M, He K (2019). Change in neutrophil to lymphocyte ratio during immunotherapy treatment is a non-linear predictor of patient outcomes in advanced cancers. J Cancer Res Clin Oncol.

[12] Jiang J, Liu R, Yu X, Yang R, Xu H, Mao Z (2019). The neutrophil-lymphocyte count ratio as a diagnostic marker for bacteraemia: a systematic review and meta-analysis. Am J Emerg Med.

[13] Yu Y, Wang H, Yan A, Wang H, Li X, Liu J (2018). Pretreatment neutrophil to lymphocyte ratio in determining the prognosis of head and neck cancer: a meta-analysis. BMC Cancer.

[14] Sainuddin S, Hague R, Howson K, Clark S (2017). New admission scoring criteria for patients with odontogenic infections: a pilot study. Br J Oral Maxillofac Surg.

[15] Zahorec R (2001). Ratio of neutrophil to lymphocyte counts-rapid and simple parameter of systemic inflammation and stress in critically ill. Bratisl Lek Listy.

[16] Rosales C (2018). Neutrophil: a cell with many roles in inflammation or several cell types?. Front Physiol.

[17] Dogruel F, Gonen Z-B, Gunay-Canpolat D, Zararsiz G, Alkan A (2017). The neutrophil-to-lymphocyte ratio as a marker of recovery status in patients with severe dental infection. Med oral Patologia oral y Cir Bucal.

[18] Pavan EP, Rocha-Junior WG, Gitt H-A, Luz JGC (2020). Changes in vital signs and laboratory tests in patients with odontogenic infections requiring hospitalization. Int J Odontostomat.

[19] Kusumoto J, Iwata E, Huang W, Takata N, Tachibana A, Akashi M (2022). Hematologic and inflammatory parameters for determining severity of odontogenic infections at admission: a retrospective study. BMC Infect Dis.

[20] Sakarozi R, Susilo DH, Wibowo MD (2022). The use of neutrophil–lymphocyte ratio (NLR) as a potential biomarker to predict the prognosis of Ludwig’s angina patients. Bali Med J.

[21] Xiaojie L, Hui L, Zhongcheng G, Chenggang W, Yaqi N. The predictive value of interleukin-6 and neutrophil-lymphocyte ratio in patients with severe and extremely severe oral and maxillofacial space infections. BioMed Research International. 2021;2021. 10.1155/2021/2615059 PMC7861909 33575324

[22] Rosca O, Bumbu BA, Ancusa O, Talpos S, Urechescu H, Ursoniu S (2022). The role of C-Reactive protein and neutrophil to lymphocyte ratio in Predicting the severity of Odontogenic Infections in adult patients. Medicina.

[23] Gallagher N, Collyer J, Bowe C (2021). Neutrophil to lymphocyte ratio as a prognostic marker of deep neck space infections secondary to odontogenic infection. Br J Oral Maxillofac Surg.

[24] Silva RdJd, Barbosa RAL, Okamura FK, Luz JGC (2023). Computed tomography analysis of fascial space involvement demonstrates correlations with laboratory tests, length of hospital stays and admission to the intensive care unit in odontogenic infections. Braz J Otorhinolaryngol.

[25] Chamora DR, Murdiyo MD, Maharani I. Association Neutrophil Lymphocyte Ratio and Therapy Respons in Submandibular Abscess with and without diabetes. Oto Rhino Laryngologica Indonesiana. 2022;51(2).

[26] Han J, Liau I, Bayetto K, May B, Goss A, Sambrook P (2020). The financial burden of acute odontogenic infections: the South Australian experience. Aust Dent J.

[27] Dogru M, Evcimik MF, Cirik AA. Is neutrophil–lymphocyte ratio associated with the severity of allergic rhinitis in children? European Archives of Oto-Rhino-Laryngology. 2016;273:3175–8. 10.1007/s00405-015-3819-y 26525883

[28] Şentürk M, Azgın İ, Övet G, Alataş N, Ağırgöl B, Yılmaz E (2016). The role of the mean platelet volume and neutrophil-to-lymphocyte ratio in peritonsillar abscesses. Braz J Otorhinolaryngol.

[29] Baglam T, Binnetoglu A, Yumusakhuylu AC, Gerin F, Demir B, Sari M (2015). Predictive value of the neutrophil-to-lymphocyte ratio in patients with deep neck space infection secondary to acute bacterial tonsillitis. Int J Pediatr Otorhinolaryngol.

[30] de Jager CP, van Wijk PT, Mathoera RB, de Jongh-Leuvenink J, van der Poll T, Wever PC (2010). Lymphocytopenia and neutrophil-lymphocyte count ratio predict bacteremia better than conventional infection markers in an emergency care unit. Crit Care.

[31] Liu X, Shen Y, Wang H, Ge Q, Fei A, Pan S. Prognostic significance of neutrophil-to-lymphocyte ratio in patients with sepsis: a prospective observational study. Mediators of inflammation. 2016;2016. 10.1155/2016/8191254 PMC4823514 27110067

[32] Velissaris D, Pantzaris N-D, Bountouris P, Gogos C (2018). Correlation between neutrophil-to-lymphocyte ratio and severity scores in septic patients upon hospital admission. A series of 50 patients. Rom J Intern Med.

[33] Wardhana AA, Lesmana T (2021). Neutrophil lymphocyte ratio (NLR) as a predictive factor radiological response of Neoadjuvant Chemotherapy (NAC) in locally advanced rectal Cancer (LARC). Bali Med J.

[34] Tanojo N, Utomo B, Ervianti E, Murtiastutik D, Prakoeswa CRS, Listiawan MY (2022). Diagnostic value of neutrophil-to-lymphocyte ratio, lymphocyte-to-monocyte ratio, and platelet-to-lymphocyte ratio in the diagnosis of erythema nodosum leprosum: a retrospective study. Trop Med Infect Disease.

[35] Akilli NB, Yortanlı M, Mutlu H, Günaydın YK, Koylu R, Akca HS (2014). Prognostic importance of neutrophil-lymphocyte ratio in critically ill patients: short-and long-term outcomes. Am J Emerg Med.

[36] Hwang SY, Shin TG, Jo IJ, Jeon K, Suh GY, Lee TR (2017). Neutrophil-to-lymphocyte ratio as a prognostic marker in critically-ill septic patients. Am J Emerg Med.

[37] Riché F, Gayat E, Barthélémy R, Le Dorze M, Matéo J, Payen D (2015). Reversal of neutrophil-to-lymphocyte count ratio in early versus late death from septic shock. Crit Care.

[38] Prabawa IPY, Bhargah A, Liwang F, Tandio DA, Tandio AL, Lestari AAW (2019). Pretreatment neutrophil-to-lymphocyte ratio (NLR) and platelet-to-lymphocyte ratio (PLR) as a predictive value of hematological markers in Cervical Cancer. Asian Pac J Cancer Prev.

